# Adherence to health regimens among frequent attenders of Finnish healthcare

**DOI:** 10.3402/ijch.v75.30726

**Published:** 2016-03-18

**Authors:** Sari Hirsikangas, Outi Kanste, Juha Korpelainen, Helvi Kyngäs

**Affiliations:** 1 Oulu University Hospital, Oulu, Finland; 2 National Institute for Health and Welfare, Oulu, Finland; 3 Medical Research Center Oulu, Oulu University Hospital, Oulu, Finland; 4 Research Group of Nursing Science and Health Management, University of Oulu, Oulu, Finland; 5 Northern Ostrobothnia Hospital District, Finland

**Keywords:** adherence, frequent attender, health regimen, chronic disease

## Abstract

**Objectives:**

The aim of the study was to describe adherence to health regimens and the factors associated with it among adult frequent attenders (FAs).

**Design:**

This was a cross-sectional study. The study sample consisted of 462 healthcare FAs in 7 municipal health centres in northern Finland. An FA is a person who has had 8 or more outpatient visits to a GP (in a health centre) or 4 or more outpatient visits to a university hospital during 1 year. The main outcome was self-reported adherence to health regimens.

**Results:**

Of the FAs, 82% adhered well to their health regimens. Carrying out self-care, medical care and feeling responsible for self-care were the most significant predictors to good adherence in all models. No significant differences in adherence were found in male and female subjects, age groups or educational levels. Support from healthcare providers and support from relatives were not significant predictors of good adherence.

**Conclusion:**

FAs in Finland adhere well to health regimens and exceptionally well to medication. Variables that predict the best adherence of FAs to health regimens are carrying out self-care, receiving medical care and feeling responsible for self-care.

Adherence to health regimens and the associated factors have been extensively studied in patients with chronic disease (1–5) but very little is known about the factors and models that predict the adherence of frequent attenders (FAs).

In this study, adherence to the health regimens of healthcare services is defined as a patient's responsible, intentional and active role in self-care, taken to maintain his or her health in collaboration with healthcare personnel (6,7). A generally accepted definition of *frequent attendance* is not available. In articles *frequent attendance* has been defined in proportional terms as a percentage (range 3–25%) of the most FAs; a commonly used definition is the 10% of patients that had the most frequent contact. The period during which contacts are counted varies from 2 to 48 months; many studies have used 12 months. The most variable problem is defining the time of day during which contacts are counted (8,9). In this study an FA refers to a person who has had 8 or more outpatient visits to a GP (in a health centre) or 4 or more outpatient visits to a university hospital during 1 year. The cut-off point of 8 visits was defined according to previous studies in Finland (10–12).

Earlier studies have shown that FAs are highly heterogeneous. They have high rates of physical disease, mental disorders and social difficulties, psychological distress, poorer health beliefs, more need of information and/or reassurance and a lower quality of life compared with non-FAs (8,12–17). Earlier studies (15,18) have shown that high use of healthcare services is often associated with hypochondria. Cross-sectional studies have shown that the somatization prevalence of FAs is high, ranging from 20 to 45% (12,14).

Illness is the main reason for frequent primary healthcare use and the roles of psychological, social and demographical factors are subordinated (19). Individual characteristics such as FA activation, psychological functioning and disease factors contribute to primary care consulting patterns among people with a chronic illness (20). FAs usually have chronic diseases that affect daily life. From the perspective of the healthcare system, the extensive use of services means a growth in demand and supply allocation needs (12).

Previous studies have shown that about half of subjects with chronic diseases have good adherence to health regimens (2,4,21,22). Earlier studies (3,23) have also indicated that factors contributing to the good adherence of subjects with chronic diseases are the following: the motivation to take care of oneself; a sense of normality; energy; and support from physicians, nurses, relatives and friends.

When collaboration with health professionals is good and patients’ treatment and care are planned together to match their normal life, patients have good adherence (24). FAs’ experiences show that healthcare staff do not understand their situations and they experience feelings of rejection and mistrust, which increases suffering (25).

In a systematic review Kripalani et al. (3) found that several types of interventions are effective in improving medication adherence in chronic medical conditions, but few were able to demonstrate an impact on clinical outcomes. Therefore the most effective interventions may be those that simplify dosing demands.

In primary care, FAs with medically unexplained symptoms often seek more emotional support from their general practitioners than do other patients, but they do not seek more explanation and reassurance or somatic intervention (26). FAs are constantly striving to be and become healthy, to be of use and to please others. FAs do not attend health centres until suffering is experienced as unbearable (25).

This cross-sectional study aims to describe the extent to which FAs adhere to health regimens and what factors predict adherence among FAs. There is no previous research on FA adherence to health regimens and factors influencing it in primary care that uses a self-report instrument on adherence in chronic disease (ACDI).

We hypothesized, based on earlier literature, that carrying out self-care, medical care, feeling responsible for self-care and support from healthcare providers or relatives would be important predictors in adherence to health regimens.

## Material and methods

### Participants

The study was conducted in 7 municipal health centres in northern Finland. The sample of healthcare FAs was collected retrospectively from the electronic medical records of municipal health centres and of Oulu University Hospital (UH) for the period 2006–2008. Patients who made at least 8 outpatient visits per year to a general practitioner (GP) in the local health centre were defined as FAs to medical services according to previous studies (10–12,14). Patients who made at least 4 outpatient visits per year to the university hospital were also defined as FAs (27). Special care was selected for inclusion because the aim was to examine the use of health services as a whole. Most FAs of hospitals indeed have primary care physicians (28,29). In order to develop healthcare services, it is important to examine the use and functionality of healthcare as a whole.

Only outpatient visits with face-to-face contacts were considered. In addition, FAs were required to be aged at least 18. Patients with visits due to pregnancy or delivery, serial treatment for the same illness, cancer palliative care, terminal hospice care, psychotic illness, mental retardation, dementia, inability to give informed consent or involved in another study intervention at the same time or just prior to this study were excluded.

Researchers identified 721 patients in the medical records of 7 health centres that met the inclusion criteria, 614 patients in primary care and 107 patients in special care. A semi-structured postal questionnaire was sent between October 2008 and May 2009. Patients were asked to give written informed consent for the study. A total of 462 questionnaires were returned (a response rate of 64%). The data do not allow analysis of the difference between those attending primary care versus special care because the number of FAs attending special care was relatively small in relation to the FAs in primary care. The aim was to research FAs as a whole.

### Data collection

The data were collected by self-report ACDI. ACDI has been shown to be a valid and reliable instrument for measuring adherence to health regimens among people with chronic illness (7,24,30). The questionnaire consisted of 38 items, with a 5-point Likert scale ranging from *strong agreement* through to *indecision* and *strong disagreement*. In this study we used the following factors: adherence to health regimens (carrying out self-care, taking medication, feeling responsible) and factors connected to adherence (support from nurses, physicians and relatives). Compliance consisted of 11 items and the scale values ranged from 0 to 55 points. A score of ≥48 points was taken to show good adherence and a score of <48 points to show poor adherence.

Other variables connected to adherence studied in this project were as follows: quality of life as measured by the 15D instrument (31), daily functioning as measured by the Frenchay Activities Index (32), sense of coherence as measured by SOC-13 (33), somatization as measured by the Symptom Checklist-90-R (34), depression symptoms as measured by Raitasalo's modification of the short form of the Beck Depression Inventory (35) and hypochondria as measured by the Whiteley Index (36). They all are valid, reliable and tested instruments. In this study we largely used the same indicators to provide information about FAs and their adherence to health regimens.

Table I shows the Cronbach's alpha coefficients for all used questionnaire items, which ranged from an acceptable to excellent level of internal consistency (α=0.67−0.93).

**Table I:** Cronbach's alpha values related to sum variables of adherence and factors connected to it

Sum variable	Number of variables	Cronbach's alpha	n
The sum variables of adherence			
Adherence to care	11	0.83	449
Carrying out self-care	6	0.81	449
Medication	3	0.85	450
Responsibility	5	0.73	450
The sum variables of factors connecting adherence			
Support			
Support from nurses	4	0.93	441
Support from physicians	4	0.89	447
Support from relatives	4	0.83	449
Somatization	12	0.67	462
Depression	13	0.86	451
Hypochondriasis	13	0.82	461
Quality of life	15	0.84	457
The activities of daily life	15	0.80	452
Sense of coherence	13	0.89	459

### Data analysis

The data were analysed with Statistical Package for the Social Sciences for Windows 17. Frequencies and percentages were used to describe the data. Cross-tabulation, the chi-square test and p-values were used to analyse the connections between variables. A Spearman's rank order correlation was run to determine the relationship between continuous variables. Factors that predicted the adherence of FAs were studied using linear regression analysis (37). Adherence was a dependent variable and we began by including the variable most highly correlated to the dependent variable in the model and kept adding explanatory variables until no further variables were significant. We used the following as independent variables: carrying out self-care; taking medication; feeling responsible for self-care; depression; hypochondriasis; somatization; daily functioning; sense of coherence; quality of life; motivation; and support from nurses, physicians and relatives. Cronbach's alpha was used to measure internal consistency.

### Ethical considerations

This study was conducted according to the ethical research guidelines of the Finnish Advisory Board on Research Integrity (2012). According to Finnish law (2010/794, 2015/143), this type of study does not need approval from an official research ethics committee. The study received administrative approval from the participating communities.

The participants were informed that the participation was voluntary, free of cost and could be interrupted at any point.

## Results

Descriptive data for our study population (n=462) are presented in Table II.

**Table II:** The characteristics of the FAs

Characteristics	n	%
Sex		
Female	301	65
Male	161	35
Age		
18–39	58	13
40–64	217	47
≥65	187	40
Education level		
Elementary education	232	51
Vocational education	217	49
Adherence to health regimens		
Good	368	82
Poor	81	18
Adherence to medication		
Yes	407	90
No	43	10
Most common chronic diseases of FAs by ICD-10		
Diseases of the musculoskeletal system and connective tissue	199	47
Diseases of the circulatory system	181	43
Diseases of the eye	119	28
Diseases of the respiratory system	107	25
Diseases of the genitourinary system	103	24
Endocrine, nutritional and metabolic diseases	89	21
Diseases of the digestive system	61	14
Diseases of the nervous system	39	9
Mental and behavioural disorders	32	8
Neoplasms	29	7

FA, frequent attender.

Two-thirds of our study population was female. The mean age was 59.4 (SD 15.1) years (range 20–95 years). Eighty-two percent of FAs reported good adherence to health regimens. The difference in adherence was not significant between male and female subjects, different age groups or educational levels. Good adherence to medication regimens was reported by 90% of FAs. One or more chronic diseases were diagnosed in 92% of FAs. The patients self-reported physician-diagnosed diseases that had continued for more than 3 months. Of those with chronic disease, 82% reported good adherence to health regimens.

Table III presents the characteristics of the FAs according to the degree of adherence. There was a statistically significant difference in adherence to health regimens between FAs whose satisfaction with life was high compared to FAs with low satisfaction (p<0.001). FAs with no somatization reported higher adherence compared to FAs with somatization (p=0.005).

**Table III:** The characteristics of the FAs with the degree of adherence to health regimens

	Degree of adherence					
			
	Good		Poor			
			
Characteristics	n	%	n	%	χ^2^	p
Sex					3.712	0.054
Female	246	85	45	15		
Male	122	77	36	23		
Age					0.419	0.811
18–39	45	79	12	21		
40–64	175	82	38	18		
≥65	148	83	31	17		
Satisfaction with life					12.192	0.001
High	305	83	62	17		
Low	53	65	28	35		
Somatization					8.006	0.005
No	332	84	64	16		
Yes	36	68	17	32		
Hypochondriasis					20.182	<0.001
No	236	89	30	11		
Yes	132	72	51	28		
Depression					4.238	0.040
None or mild	312	84	61	16		
Moderate or severe	56	74	20	26		
Adherence to medication					159.094	<0.001
Yes	363	89	43	11		
No	5	12	38	88		

Hypochondriacal beliefs were more common among FAs who had poor adherence to health regimens compared to FAs with good adherence (p<0.001). FAs with no or mild depression reported better adherence compared to FAs with moderate or severe depression. FAs with good adherence to health regimens also showed good adherence to medication regimens.

There was a slight positive correlation between adherence to health regimens and daily functioning (r=0.122, p=0.015). A positive correlation was also found between adherence and quality of life (r=0.210, p<0.001) and between adherence and sense of coherence (r=0.232, p<0.001).

Table IV compares independent variables that predict the adherence of FAs to health regimens in a linear regression. Independent variables were found to be as follows: carrying out self-care, taking medication, feeling responsible for care, support from nurses, support from relatives, depression symptoms, hypochondriasis, somatization, daily functioning, sense of coherence and quality of life. The best multiple linear regression models were Model 8 and Model 9. For the 3 explanatory variables, both models had an adjusted R-squared value of 0.92, which indicated a very good fit for the model.

**Table IV:** Variables that predict adherence in linear regression

	Standardized coefficients and p-values									
	
Variables	Univariate	Model 1	Model 2	Model 3	Model 4	Model 5	Model 6	Model 7	Model 8	Model 9
Carrying out self-care	0.901						0.894 (<0.001)	0.896 (<0.001)		
Medication	0.789		0.779 (<0.001)	0.764 (<0.001)					0.547 (<0.001)	0.547 (<0.001)
Responsibility for self-care	0.815	0.763 (<0.001)			0.784 (<0.001)	0.805 (<0.001)			0.589 (<0.001)	0.589 (<0.001)
Support from nurses	0.252			0.127 (<0.001)						
Support from relatives	0.283	0.083 (0.010)			0.064 (0.035)					
Depression	−0.149									−0.035 (0.017)
Hypochondriasis	−0.176		−0.064 (0.061)	−0.070 (0.027)			−0.043 (0.048)			
Somatization	−0.132		−0.065 (0.056)					−0.059 (0.006)		
Daily functioning	0.105	0.068 (0.027)								
Sense of coherence	0.178					0.069 (0.017)				
Quality of life	0.153				0.062 (0.036)				0.035 (0.017)	
R^2^		0.63	0.63	0.65	0.66	0.67	0.81	0.81	0.92	0.92

Univariate: the relationship between dependent variables (adherence) and independent variables; standardized coefficients beta value. Model 1: statistically significant variables: responsibility for self-care, support from relatives and daily functioning. Model 2: statistically significant variables: medication, hypochondriasis and somatization. Model 3: statistically significant variables: medication, support from nurses and hypochondriasis. Model 4: statistically significant variables: responsibility for self-care, support from relatives and quality of life. Model 5: statistically significant variables: responsibility for self-care and sense of coherence. Model 6: statistically significant variables: carrying out self-care and hypochondriasis. Model 7: statistically significant variables: carrying out self-care and somatization. Model 8: statistically significant variables: medication, responsibility for self-care and quality of life. Model 9: statistically significant variables: medication, responsibility for self-care and depression.

Carrying out self-care, taking medication and feeling responsible for self-care were the most significant predictors of good adherence with health regimens in all models. When an FA adhered to the medication regimen well and reported having a high level of responsibility for his or her self-care and a good quality of life, the coefficient of the determination of adherence was optimal (Model 8). The coefficient of the determination of quality of life was low compared to medication and feeling responsible for self-care in Model 8. Support from nurses, physicians and relatives were not relevant predictors of good adherence. Depression, hypochondriasis, somatization, activities of daily living and sense of coherence were not significant predictors in any model.

## Discussion

This study presents for the first time the results of a self-report ACDI on FA adherence to health regimens and the factors infiuencing adherence in primary care in Finland. The key findings of this study were that FAs adhered well to health regimens. Life satisfaction positively affected adherence. The most significant factors predicting adherence among FAs were carrying out self-care, taking medication and feeling responsible for self-care. As well this study showed that good daily functioning, good quality of life and a high sense of coherence had a slight connection with good adherence to health regimens. Based on an earlier study (38), a sense of coherence has significant influence on frequent attendance in primary healthcare.

In total, 82% of FAs reported good adherence to health regimens. By comparison, earlier studies focusing on adherence of chronically ill patients to health regimens indicated that about half to two-thirds of such patients had good adherence (2,5,21,22,39). Based on our understanding in this study, FAs with one or more chronic disease seemed to be more adherent to health regimens, possibly resulting from an increased sense of safety related to adherence. One of the most important aspects of FA well-being and daily activities is that the person takes care of her or his health well. The average age of FAs was about 60 and they had many chronic diseases. Their own actions have a big impact on their well-being in the future. FAs may fear that their illnesses will have adverse effects on their life, and this fear may be sufficient motivation for good adherence. Patients are able to assume full responsibility for their care if they have the necessary knowledge and skills as well as the requisite mental readiness (5).

More than 90% of FAs had one or more chronic disease. The most common diseases were diseases of the musculoskeletal system and connective tissue and diseases of the circulatory system. This finding is in line with earlier studies (12,40,41). People with chronic diseases require more services due to characteristics that increase their vulnerability (42). Poor adherence in the treatment of chronic diseases is a problem. The consequences of poor adherence to long-term therapies are poor health outcomes and increased healthcare costs. Interventions aimed at increasing the effectiveness of adherence may have a far greater impact on the population's health than any improvement in specific medical treatments (43).

FA adherence to medication regimens was 90%. Previous studies have shown that the overall rate of adherence to medications for patients with chronic diseases was 36–97% (24,44,45). FAs generate 5 times as many prescriptions compared to less FAs (46). FAs receive more prescriptions for anxiolytics, analgesics, sleeping tablets, antidepressants and antibiotics compared with normal attenders (9). They have to comply with several medications in order to keep their chronic diseases under control and their quality of life good. If FAs are made well aware of the consequences of failure to follow medication regimens, they know why they have to follow the instructions.

This study showed that age, sex and education had no significant association with the level of adherence to a regimen. An earlier study (5) showed that most patients with glaucoma who adhered well were found in the 62–73 year-old age group. In contrast, another study (24) showed that patients on warfarin therapy aged 27–65 years had better adherence than others and females had better adherence than males. There is insufficient evidence of the influence of age and sex on the level of adherence.

Likewise, in our study support from healthcare providers or relatives was not a significant predictor for good adherence. This result is the opposite of findings from previous studies. There is evidence that support from healthcare providers leads to good adherence (24,47,48). This study result may be explained by the fact that the patients in this study were FAs of healthcare services. They had a great deal of contact with healthcare providers and did not need further special support from them.

Among all FAs, the prevalence of hypochondriasis was 41%, though it was slightly lower among FAs with good adherence (36%). Previous studies have shown similar results, ranging from 36 to 50% (12,14). Earlier studies (6,49) have indicated an association between a high score in hypochondriasis and high use of healthcare services. FAs use services frequently for increasingly complex health needs (50). They have less sense of coherence (38) and greater health anxiety and hypochondriacal beliefs compared to other attenders (14,15). They need healthcare providers in order to get an explanation for their symptoms and to cope with them.

The prevalence of somatization among FAs with good adherence was 8%, whereas among all FAs it was 12%. This result differs from previous studies (12,14) that reported a somatization prevalence among FAs ranging from 20 to 45%. An earlier study (6) showed a causal relationship between psychological distress and frequent attendance. Whether low somatization is the result of good adherence of the FAs in this study. FAs receive healthcare assistance to meet their needs on time because they make heavy use of medical services. The prevalence of mental and behavioural disorders was not significant (7.6%) in this study compared to an earlier study (36%) (12). It may be also the result of good adherence and well-being.

According to the linear regression, variables that predicted the best adherence of FAs to health regimens in every model were as follows: carrying out self-care, receiving medical care and feeling responsible for self-care. They were the most important single predictors in relation to other variables. According to previous studies (3,7,23,51), factors contributing to good adherence among patients with chronic diseases are motivation to take care of oneself; support from physicians, nurses, relatives and friends; a sense of normality; and energy. In this study we assumed that when FAs adhered well to their medication it produced a sense of normality. They received support from healthcare providers by using medical services actively, which contributes to good adherence.

On the basis of our results, FAs may benefit from a case management approach to meeting their needs and to promoting adherence to health regimens. Based on our understanding it seems that FAs with chronic diseases and those who mainly suffer from hypochondriasis need regular, anticipatory care with a holistic approach. A case management nurse (who is a specialized registered nurse) would enable effective counselling based on individual needs and provide support to improve the well-being of FAs. The case management nurse would help them navigate the healthcare system (52).

This study has many strengths. The first is the novelty of the research area; this topic has not been studied previously. Another is the high response rate (62%). The third strength is that the sample of FAs was carefully selected; the evidence that they were FAs is strong because that information was collected from medical records. Finally, the instruments used in the study are commonly used and their validity and reliability were found to be high. However, there was one limitation: the instruments were self-reported and it is known that social desirability bias is always present in self-reported responses.

## Conclusions

This study indicates that FAs in healthcare in Finland adhere well to health regimens and exceptionally well to medication regimens. The variables that predicted the best adherence of FAs to health regimens in this study were carrying out self-care, receiving medical care and feeling responsible for self-care. The findings of this study will be useful in developing an intervention using case management and other support methods for FAs with the aim of improving adherence to health regimens. In our next study we will evaluate the effectiveness of such an intervention and the factors that contribute to better adherence to health regimens.
